# Comparison of Guideline‐Directed Medical Therapy in Black versus White Patients With Heart Failure With Reduced Ejection Fraction in a Large, Integrated Health Care System

**DOI:** 10.1002/clc.70452

**Published:** 2026-08-30

**Authors:** Natalia C. Berry, Yi‐Shin Sheu, Karen Chesbrough, R. Clayton Bishop, Suma Vupputuri

**Affiliations:** ^1^ Department of Cardiology Mid‐Atlantic Permanente Medical Group McLean Virginia USA; ^2^ Mid‐Atlantic Permanente Research Institute, Mid‐Atlantic Permanente Medical Group Washington District of Columbia USA

**Keywords:** guideline‐directed medical therapy, heart failure with reduced ejection fraction, racial differences, real‐world population

## Abstract

**Background:**

Guideline‐directed medical therapy (GDMT) improves clinical outcomes for patients with heart failure with reduced ejection fraction (HFrEF). While GDMT treatment differences have been described for Black patients, data are limited.

**Aims:**

We sought to evaluate, among Black and White HFrEF patients, differences in GDMT use and target dosing by race.

**Materials & Methods:**

We examined health records of Black and White patients with HFrEF between 2015 and 2022. GDMT was defined by four medication classes—ACEi/ARB/ARNi, beta blocker (BB), mineralocorticoid receptor antagonist (MRA), and SGLT2i. Proportions of patients on GDMT and achieving target dose were examined for each class. Logistic regression was used to assess variables associated with differential GDMT use and dosing.

**Results:**

Our cohort totaled 2825 HFrEF patients (61.2% Black; 38.8% White). Compared to White patients, Black patients had higher treatment with ACEi/ARB/ARNi (84.1% vs. 80.0%), BB (85.3% vs. 74.9%), and MRA (27.9% vs.18.4%), but similar SGLT2i treatment. Among treated, Black patients were more likely to be treated to target with ACEi/ARB/ARNi (45.2% vs. 34.2%), BB (25.0% vs. 17.8%), and MRA (80.3% vs. 72.1%). After multivariate adjustment, MRA treatment was associated with race (OR = 1.39; 95% CI: 1.10–1.75). Among treated, Black patients had higher odds of treatment to target with ACEi/ARB/ARNi (OR = 2.0; 95% CI: 1.61–2.53) and MRA (OR = 1.6; 95% CI: 1.01–2.57).

**Conclusions:**

Using real‐world data from a diverse health system, we demonstrated that Black patients had 2.0 times and 1.6 times greater odds of being treated to target with ACEi/ARB/ARNi and MRAs, respectively, compared to White patients. Future research should explore whether these differences translate to improved health outcomes.

## Graphical abstract

Using real‐world data, this study identified differences in recommended GDMT treatment between Black and White HFrEF patients. Black patients were more likely to be treated with MRAs than White patients. Among those treated with GDMT, Black patients were more likely to be treated to target with ACEi/ARB/ARNis and MRAs.

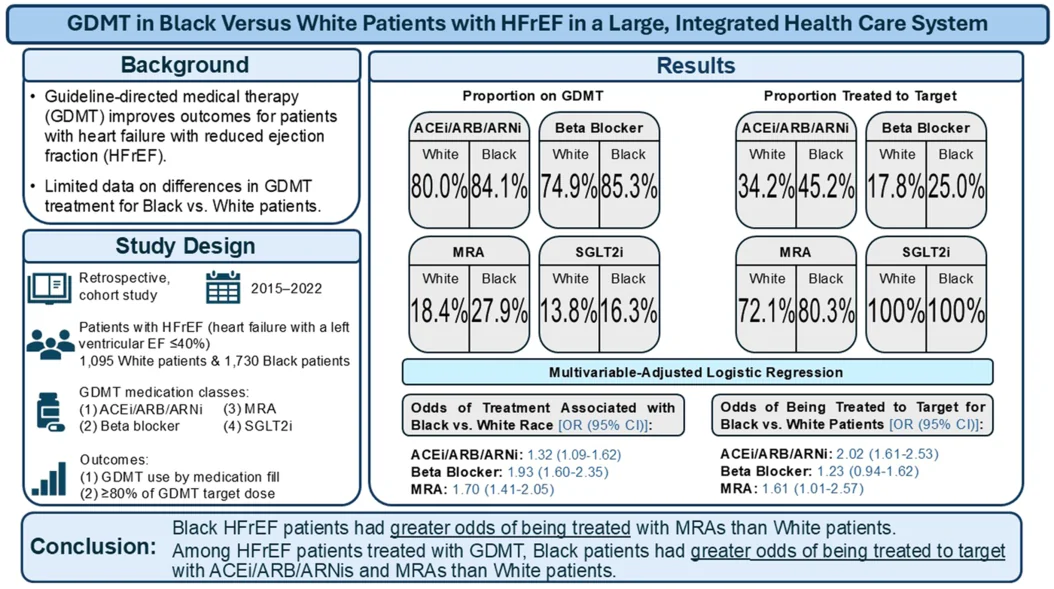

**Abbreviations**
ACEi: angiotensin‐converting enzyme inhibitor
ARB: angiotensin receptor blockers
ARIC: Atherosclerosis Risk in Communities (ARIC)
ARNi: angiotensin receptor‐neprilysin inhibitor
BB: beta blockers
GDMT: guideline‐directed medical therapy (GDMT)
GFR: glomerular filtration rate
HFrEF: heart failure with reduced ejection fraction
MRA: mineralocorticoid receptor antagonist
NDI: neighborhood deprivation index (NDI)
PCP: primary care physician
SGLT2i: sodium‐glucose transport protein 2 inhibitor

## 1 Introduction

Heart failure with reduced ejection fraction (HFrEF), defined as heart failure (HF) with a left ventricular ejection fraction ≤ 40%, presents a significant burden to patients and society at large, affecting nearly 6.5 million American adults and with greater than 1 million hospitalizations annually [1, 2]. While guideline‐directed medical therapy (GDMT) is a Class 1 recommendation for the treatment of HFrEF by the American Heart Association and American College of Cardiology [1, 3], the uptake of GDMT and its components remains suboptimal [4]. Prior studies have reported racial differences in HF trends—namely that Black patients are diagnosed with HF at younger ages [5], are more likely to develop HF, and also have higher risks of HF hospitalization and HF‐related death compared with non‐Black counterparts [6, 7, 8]. Data from the Get With the Guidelines Registry demonstrated that neighborhood socioeconomic disadvantage disproportionately affected Black patients and was associated with higher in‐hospital mortality [9], and contributed more significantly to a prolonged length of stay [10]. Prior studies have noted a higher proportion of optimal GDMT among Black patients, and higher rates and target dosing of Black patients within individual GDMT classes [11, 12]. However, differences between Black and White patients in GDMT use in real‐world HF populations in the United States remain poorly understood.

We conducted a retrospective cohort study using comprehensive patient electronic health records (EHR) from a large, diverse health system. The objectives of our study were, among patients with HFrEF within the Kaiser Permanente Mid‐Atlantic States (KPMAS) health system, to assess: (1) racial differences between Black and White patients in the proportion of patients taking four medication classes of GDMT; (2) racial differences in the proportion of patients receiving the recommended target dose of GDMT medication classes; and (3) the effect of Black race on both GDMT treatment use and target dosing for each class of medication.

## 2 Methods

### 2.1 Study Design and Population

This retrospective, cohort study of KPMAS patients with HF was conducted between January 1, 2015 and December 31, 2022 [13]. HF was defined using a previously validated algorithm using one or more hospitalizations with a primary diagnosis of HF and/or having ≥ 3 ambulatory visits coded for HF (ICD codes are provided in the Supplemental Methods) [14, 15, 16]. LVEF was assessed using results from echocardiogram or other imaging modalities and HFrEF was defined as an LVEF ≤ 40%. The study index date was defined as the date of the first occurrence of LVEF ≤ 40%.

The inclusion criteria for our study were patients: (1) with diagnosed HF and LVEF ≤ 40%; (2) aged ≥ 18 years of age at index; (3) of Black or White race; and (4) with 12 months of continuous enrollment prior to index date. Excluded patients included those with history of end‐stage renal disease, heart transplantation, left ventricular assist device, hospice status indicated within the EHR prior to the index date, and non‐KPMAS patients. In addition, patients were excluded from the analysis if they were pregnant at the time of index date, died within 1 month of the index date, or if they had missing medication data. A subset of the cohort was developed to specifically assess SGLT2i. This cohort was limited to HF patients with index dates between January 1, 2020 and December 31, 2022, given that the major clinical trials supporting SGLT2i use in HFrEF were published in 2019–2020 [17, 18].

### 2.2 Study Variables

Patient measures were extracted from the EHR and assessed during the 36‐month period preceding the index date (Supporting Information S1: Table S1). In instances of multiple measures of continuous variables per patient, the mean for an individual was calculated. In instances of multiple measures of categorical variables, the value closest to index was utilized. Race was self‐reported. Neighborhood deprivation index (NDI) is a composite score that empirically summarizes neighborhood disadvantage. It was derived from multiple census tract level indicators of neighborhood deprivation including income/poverty, education, employment, and occupation [19]. History of comorbidities and cardiac procedures was ascertained through the presence or absence of corresponding ICD‐9/10 and CPT codes.

### 2.3 Study Outcomes

Medication fill data for four classes of GDMT were obtained during the 12 months following the index date. The medication classes included: angiotensin‐converting enzyme inhibitor/angiotensin receptor blockers/angiotensin receptor‐neprilysin inhibitors (ACEi/ARB/ARNis), beta blockers (BB), mineralocorticoid receptor antagonists (MRAs), sodium‐glucose transport protein 2 inhibitors (SGLT2is). The two outcome measures, assessed separately for each GDMT medication class, were: (1) GDMT medication class use defined by medication fills; and (2) a dosing regimen achieving at least 80% of the target dose for each of the GDMT classes. The percentage of target dose was calculated by dividing the average daily dose per patient by the established target dose for a given medication class and weighted by the number of days on that medication. Within each class of medication, patients not taking the medication were excluded from the target dose analysis. Established target doses by medication are detailed further in Supporting Information S1: Table S2. In the target dose analysis, we chose a percentage of target dose threshold of ≥ 80% to define target dose. This threshold was chosen based on mean doses achieved in the majority of clinical trials for GDMT, as well as to allow for a period of uptitration in the weighted average dose and to reflect realistic prescribing patterns and patient adherence in real‐world practice [20].

### 2.4 Statistical Analysis

Continuous variables were reported as means and standard deviations (SDs), while categorical variables were summarized using frequencies and percentages. Missing data was not a significant concern; the variables with the most missing data were NDI quartile (8 patients or 0.3%), and glomerular filtration rate (GFR; 76 patients, or 2.4%) for demographic and standard laboratory values, respectively.

Logistic regression models were used to assess associations of race with GDMT use and achieving target dose. Models included covariates that were potential confounders and were presented sequentially adjusted based on convenience groupings. Models presented include: (1) unadjusted, (2) adjusted for age and sex, (3) further adjusted by NDI, (4) further adjusted by clinical characteristics, (5) further adjusted by utilization. Unadjusted and adjusted odds ratios (ORs) were reported with 95% confidence intervals (CI). Patients with a contraindication to a particular class of GDMT (contraindications are listed by GDMT class in Supporting Information S1: Table S3), were excluded from the logistic regression models. All data analyses were conducted using Python 3.10 with statsmodels 0.14.1 [21, 22], Pandas 2.2.1 [23], Matplotlib 3.8.4 [24], and Seaborn 0.13.2 [25] packages. *p*‐values less than 0.05 were considered statistically significant.

This study was approved by the KPMAS Institutional Review Board with a waiver of informed consent.

## 3 Results

### 3.1 Cohort Characteristics

**Figure 1:**
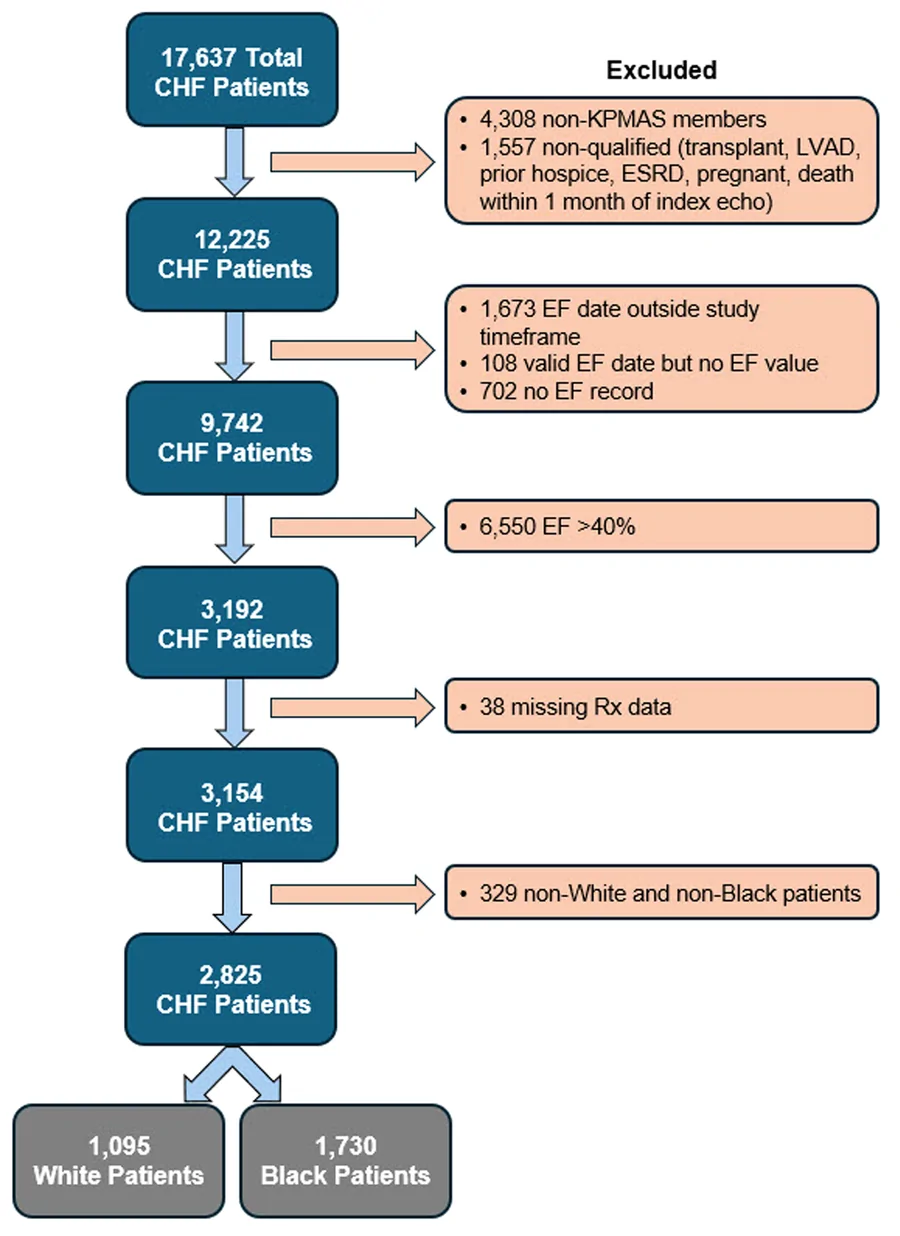
Study consort diagram.

Our final study sample totaled 2825 HFrEF patients after inclusion and exclusion criteria were applied (Figure 1). The cohort was comprised of 1730 (61.2%) Black patients and 1095 (38.8%) White patients. In comparison with White patients, Black patients were on average 6.9 years younger at index, less likely to be male, and more likely to be commercially insured (Table 1). The greatest proportion of Black patients lived in the most deprived neighborhoods (44.5% vs. 10.5% White, *p* < 0.001), whereas the greatest proportion of White patients lived in the least deprived neighborhoods (37.3% vs. 10.1% Black, *p* < 0.001). Many comorbidities were less common among Black patients compared to White patients, including dyslipidemia or hyperlipidemia (*p* < 0.001), coronary artery disease (*p* < 0.001), atrial fibrillation/flutter (*p* < 0.001), and chronic obstructive pulmonary disease (COPD) (*p* = 0.016). However, asthma (*p* = 0.047), tobacco use (*p* = 0.048), elevated hemoglobin A1C (*p* = 0.009), and reduced kidney function (*p* < 0.001) were more common in Black patients than in White patients. The proportion of patients with a history of hypertension and ischemic stroke/TIA were similar. Black patients had higher mean body mass index (*p* < 0.001), systolic blood pressure (*p* < 0.001), heart rate (*p* < 0.001), and lower mean index LVEF (*p* < 0.001) compared to White patients. Black patients demonstrated lower healthcare utilization, with fewer cardiology visits (*p* < 0.001) and fewer inpatient hospitalization (*p* = 0.003) than White patients.

**Table 1:** Characteristics of HFrEF study sample, by race.

Characteristics	White patients (*N* = 1095)	Black patients (*N* = 1730)	*p*‐value
**Demographics**			
Age at index date, mean (SD), year	71.7 (12.1)	64.8 (12.7)	< 0.001
Sex, *n* (%)			
Male	775 (70.8)	1062 (61.4)	< 0.001
Female	320 (29.2)	668 (38.6)	
Neighborhood deprivation index, *n* (%)			
1st Quartile	486 (44.5)	181 (10.5)	< 0.001
2nd Quartile	294 (26.9)	404 (23.4)	
3rd Quartile	202 (18.5)	498 (28.9)	
4th Quartile	110 (10.1)	643 (37.3)	
**Clinical characteristics**			
Left ventricular ejection fraction, mean (SD)	31.4 (7.5)	28.9 (8.2)	< 0.001
Medical history, *n* (%)			
History of atrial fibrillation or flutter	528 (48.2)	464 (26.8)	< 0.001
History of ischemic stroke or transient ischemic attack	389 (35.5)	670 (38.7)	0.094
History of coronary artery disease	684 (62.5)	798 (46.1)	< 0.001
Hypertension	897 (81.9)	1443 (83.4)	0.330
Dyslipidemia/hyperlipidemia	810 (74.0)	1128 (65.2)	< 0.001
Chronic liver disease/cirrhosis	125 (11.4)	235 (13.6)	0.104
Asthma	161 (14.7)	305 (17.6)	0.047
Chronic obstructive pulmonary disease	390 (35.6)	539 (31.2)	0.016
Home oxygen therapy	58 (5.3)	42 (2.4)	< 0.001
Depression	271 (24.8)	260 (15.0)	< 0.001
Dementia/cognitive conditions	160 (14.6)	230 (13.3)	0.351
Smoker	433 (39.5)	619 (35.8)	0.048
Vital signs			
Systolic blood pressure, mean (SD), mmHg	128.5 (13.6)	133.9 (14.9)	< 0.001
Body mass index, mean (SD), kg/m^2^	30.4 (6.8)	32.2 (7.9)	< 0.001
Heart rate, mean (SD), bpm	78.4 (11.3)	80.8 (10.7)	< 0.001
Laboratory results			
Hemoglobin A1C, *n* (%)			
High (≥ 6.5%)	322 (29.4)	597 (34.5)	0.009
Elevated (5.7%–6.4%)	263 (24.0)	402 (23.2)	
Normal (< 5.7%)	177 (16.2)	219 (12.7)	
Unknown	333 (30.4)	512 (29.6)	
Estimated glomerular filtration rate (eGFR), *n* (%)			
Severe (< 30 mL/min/1.73 m^2^)	32 (2.9)	93 (5.4)	< 0.001
Moderate (30–60 mL/min/1.73 m^2^)	284 (25.9)	582 (33.6)	
Normal (> 60 mL/min/1.73 m^2^)	750 (68.5)	1020 (59.0)	
Unknown	29 (2.7)	35 (2.0)	
**Utilization**			
Encounters, 12‐month period, median (IQR)			
Cardiology visits	7 (16)	5 (12)	< 0.001
Primary care visits	1 (23)	0 (20)	0.057
Inpatient hospitalizations	0 (0)	0 (0)	0.003
Inpatient hospitalizations, 12‐month period, *n* (%)			
0	906 (82.7)	1352 (78.1)	0.011
1–2	172 (15.7)	349 (20.2)	
3+	17 (1.6)	29 (1.7)	
Automated implantable defibrillator implant, *n* (%)	127 (11.6)	179 (10.4)	0.327

### 3.2 GDMT Use and Target Dosing

**Figure 2:**
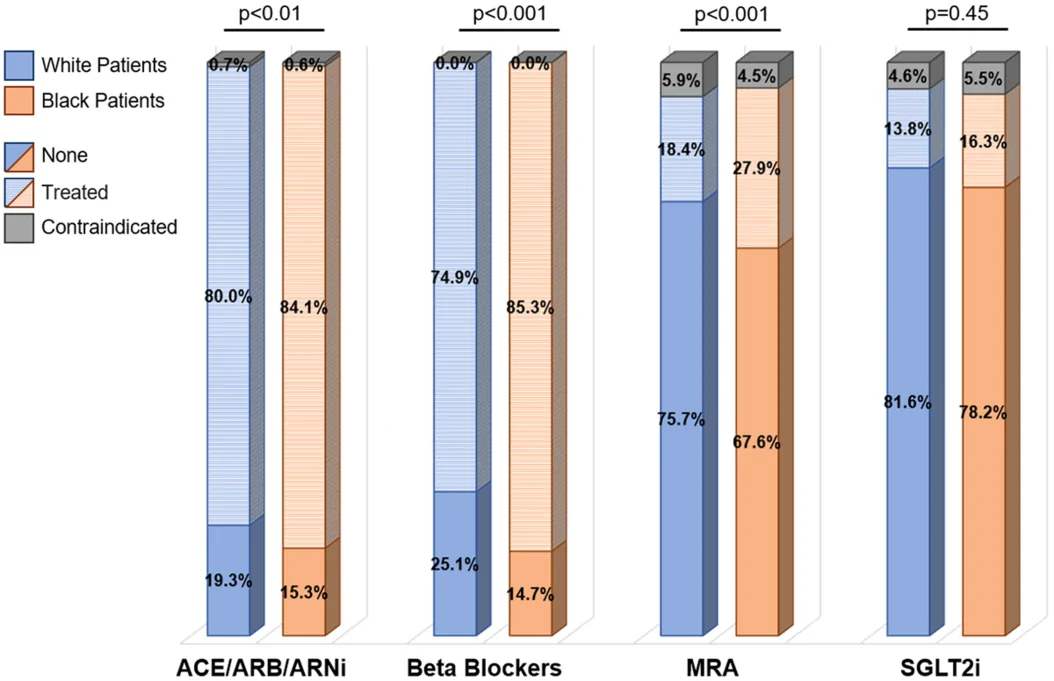
Proportion of patients with HFrEF using GDMT medications classes by race. Patients with contraindication for each class of GDMT numbered 8, 0, 65, 11 for White patients, and 11, 0, 77, 19 for Black patients for ACE/ARB/ARNi, beta blocker, MRA, SGLT2i, respectively. Data for SGLT2i were calculated from a subset of the cohort restricted to HFrEF patients with index dates between January 1, 2020 and December 31, 2022 [344 (59.0%) Black patients and 239 (41.0%) White patients].

**Figure 3:**
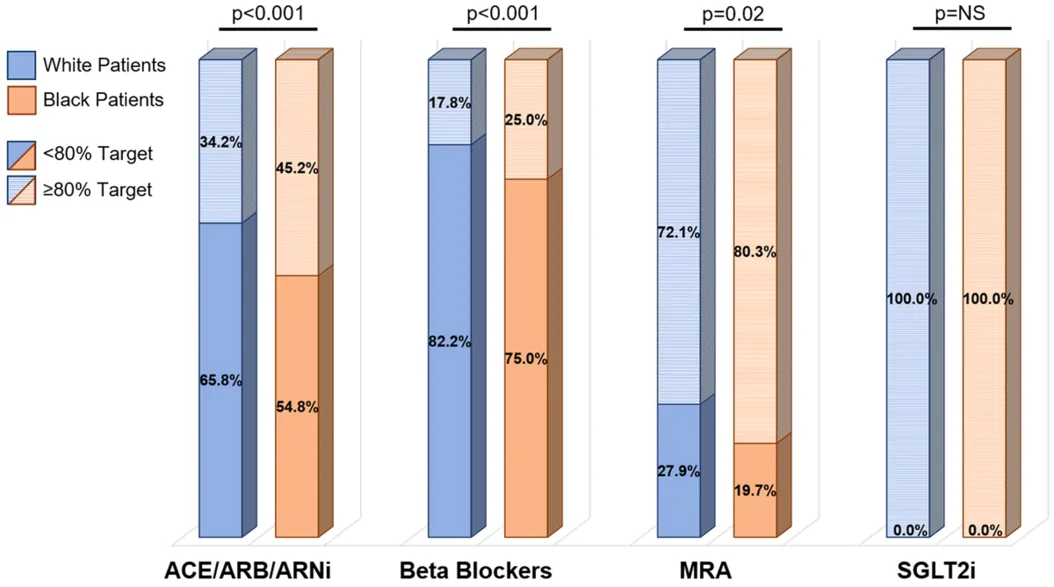
Proportion of patients with HFrEF treated to target (≥ 80%) with GDMT medication classes, by race.

In total, 1661 (96.0%) Black patients and 997 (91.1%) White patients were on some form of GDMT, respectively. For each medication class, the percentage of patients treated, not treated, and with a medication class contraindication are displayed in Figure 2. Patients with a contraindication for each class of GDMT numbered 8, 0, 65, 11 for White patients, and 11, 0, 77, 19 for Black patients for ACEi/ARB/ARNi, BB, MRA, SGLT2i, respectively. Black patients had significantly higher treatment rates than White patients with ACEi/ARB/ARNi (84.1% vs. 80.0%, *p* < 0.01), BB (85.3% vs. 74.9%, *p* < 0.001), and MRA (27.9% vs.18.4%, *p* < 0.001). Rates of SGLT2i treatment were similar between groups (16.3% vs. 13.8%, *p* = 0.45). Among those treated with GDMT, the percentages of patients treated with < 80% of target dose and ≥ 80% of target dose are reported in Figure 3. Black patients were more likely to be treated at ≥ 80% of target dose than White patients for ACEi/ARB/ARNi (45.2% vs. 34.2%, *p* < 0.001), BB (25.0% vs. 17.8%, *p* < 0.001), and MRA (80.3% vs. 72.1%, *p* = 0.02).

### 3.3 Associations of Race With GDMT Use

Estimates from logistic regression models reported the association between race and GDMT use. In crude models, we observed significantly higher odds of treatment for Black versus White patients with ACEi/ARB/ARNi (OR = 1.32; 95% CI: 1.09–1.62), BB (OR = 1.93; 95% CI: 1.60–2.35), and MRA (OR = 1.70; 95% CI: 1.41–2.05) (Table 2). After full covariate adjustment, race was no longer independently associated with the use of ACEi/ARB/ARNi or BB (all covariate ORs presented in Supporting Information S1: Table S4). However, for the MRA class, the higher odds of treatment among Black versus White patients persisted after adjustment for age, sex, NDI, clinical comorbidities/characteristics, and utilization metrics (OR = 1.39; 95% CI: 1.10–1.75).

**Table 2:** Associations of race with use of GDMT medication classes among patients with HFrEF.

	GDMT class use		
	ACEi/ARB/ARNi	BB	MRA
Logistic models	OR (95% CI)	OR (95% CI)	OR (95% CI)
Including race	1.32 **(1.09, 1.62)**	1.93 **(1.60, 2.35)**	1.70 **(1.41, 2.05)**
Including race age, sex	1.11 (0.90, 1.37)	1.50 **(1.23, 1.83)**	1.41 **(1.16, 1.71)**
Including race age, sex, NDI	1.17 (0.93, 1.48)	1.43 **(1.15, 1.79)**	1.32 **(1.07, 1.63)**
Including race, age, sex, NDI + comorbidities/clinical characteristics^a^	1.13 (0.87, 1.46)	1.21 (0.95, 1.55)	1.38 **(1.10, 1.75)**
Including race, age, sex, NDI + comorbidities/clinical characteristics + utilization^b^	1.14 (0.88, 1.47)	1.20 (0.94, 1.53)	1.39 **(1.10, 1.75)**

*Note:* **Bolded** 95% confidence intervals indicate statistical significance.

^a^ **Comorbidities/clinical characteristics** = history of atrial fibrillation/flutter, stroke/transient ischemic attack, coronary artery disease, hypertension, hyperlipidemia, chronic liver disease/cirrhosis, asthma, chronic obstructive pulmonary disease, home oxygen therapy, depression, dementia, left ventricular ejection fraction, smoking, body mass index, systolic blood pressure, estimated glomerular filtration rate (categorical), hemoglobin A1C (categorical).

^b^ **Utilization** = cardiology encounters, PCP encounter, heart failure related hospitalization within 12 months, defibrillator.

### 3.4 Associations of Race With GDMT Target Dosing

Logistic regression models were also constructed to assess association of race and achieving ≥ 80% of GDMT target dosing (Table 3). In unadjusted models, we observed higher odds of achieving target medication dose among Black versus White patients for ACEi/ARB/ARNi (OR = 2.33; 95% CI: 1.96–2.77), BB (OR = 1.54; 95% CI: 1.24–1.91), and MRA (OR = 1.58; 95% CI: 1.08–2.31). After full covariate adjustment, race was no longer significantly associated with achieving target medication dose of BB (OR = 1.23; 95% CI: 0.94–1.62) (all covariate ORs presented in Supporting Information S1: Table S5). However, associations between race and achieving GDMT target dose for ACEi/ARB/ARNi (OR = 2.02; 95% CI: 1.61–2.53) and MRA (OR 1.61; 95% CI: 1.01–2.57) remained significant, though their magnitude was attenuated compared to the unadjusted model.

**Table 3:** Associations of race with target treatment (≥ 80%) of GDMT medication classes among patients with HFrEF.

	GDMT class target treatment		
	ACEi/ARB/ARNi	BB	MRA
Logistic models	OR (95% CI)	OR (95% CI)	OR (95% CI)
Including race	2.33 **(1.96, 2.77)**	1.54 **(1.24, 1.91)**	1.58 **(1.08, 2.31)**
Including race adjusted for age, sex	2.35 **(1.97, 2.82)**	1.40 **(1.13, 1.75)**	1.59 **(1.07, 2.35)**
Including race adjusted for age, sex, NDI	2.24 **(1.84, 2.72)**	1.32 **(1.03, 1.67)**	1.69 **(1.11, 2.57)**
Including race adjusted for age, sex, NDI + comorbidities/clinical characteristics^a^	2.03 **(1.62, 2.54)**	1.25 (0.96, 1.63)	1.61 **(1.01, 2.56)**
Including race adjusted for age, sex, NDI + comorbidities/clinical characteristics + utilization^b^	2.02 **(1.61, 2.53)**	1.23 (0.94, 1.62)	1.61 **(1.01, 2.57)**

*Note:* **Bolded** 95% confidence intervals indicate statistical significance.

^a^ **Comorbidities/clinical characteristics** = history of atrial fibrillation/flutter, stroke/transient ischemic attack, coronary artery disease, hypertension, hyperlipidemia, chronic liver disease/cirrhosis, asthma, chronic obstructive pulmonary disease, home oxygen therapy, depression, dementia, left ventricular ejection fraction, smoking, body mass index, systolic blood pressure, estimated glomerular filtration rate (categorical), hemoglobin A1C (categorical).

^b^ **Utilization** = cardiology encounters, PCP encounter, heart failure related hospitalization within 12 months, defibrillator.

## 4 Discussion

We observed in our analysis of a large, diverse patient cohort with HFrEF that Black patients demonstrated significantly higher odds of being treated with MRAs (but not other GDMT medication classes) than White patients. Among GDMT‐treated patients, Black patients demonstrated 2.0 times greater odds of achieving the target dose of ACEi/ARB/ARNi and 1.6 times greater odds of achieving the target dose of MRAs than White patients.

Our findings reflect similarity to prior studies showing higher rates of optimal GDMT prescription in Black HFrEF patients compared with White patients [11, 26, 27]. The Atherosclerosis Risk in Communities (ARIC) study reported longitudinal HF events in communities across four states over 9 years, and demonstrated higher rates of ACEi/ARB, BB, and MRA treatment in Black patients [11]. They furthermore noted that Black patients were more likely to be on optimal (defined as ACEi/ARB, BB, and MRA) or acceptable (defined as two out of three therapies) GDMT than White patients. OPTIMIZE‐HF reported higher rates of ACEi and MRA prescription among Black patients compared with non‐Black patients at hospital discharge, and after adjustment, demonstrated an independent association between Black race and ACEi prescription (although no measure of socioeconomic status was accounted for) [28]. BB prescriptions were not noted to be different between Black and non‐Black patients. In CHAMP‐HF, Black race was associated with a higher likelihood of treatment to target with ACEis and BBs [4]. Finally, in a recent report of predominantly male Veterans Affairs patients with HFrEF, Black patients were shown to have significantly higher rates of BB, ACEi/ARB, and MRA treatment than White patients [12].

Compared with CHAMP‐HF and OPTIMIZE‐HF, which are prospective registries with voluntary participation, and the Veterans Affairs data, which was predominantly male, we believe that our study offers novel insight into real‐world differences among Black and White patients and GDMT rates and dosing. Black patients outnumbered White patients in our population, which is unique and adds to the robustness of our conclusions about differential treatment and target treatment rates. Additionally, the longitudinal and detailed nature of our EHR facilitated a detailed examination of confounding factors (including healthcare utilization), which is another unique aspect to our study.

We observed Black patients to be younger than White patients in our cohort, which could potentially explain the higher proportion of Black patients being treated and treated to target across three GDMT medication classes in the unadjusted model. Younger patients may present with clinical profiles and fewer contraindications that make them more suitable candidates for GDMT therapy. Furthermore, our data revealed that Black patients had higher systolic blood pressure and resting heart rate, likely rendering them better candidates for treatment with and uptitration of GDMT medications. Indeed, prior studies have hypothesized that higher treatment rates with GDMT medications observed in Black patients may be due to higher prevalence of hypertension, diabetes, and lower EFs in this population [4, 11], which we also observed in our study.

After adjusting for demographic and clinical characteristics, however, race did not independently predict ACEi/ARB/ARNi nor BB utilization. We did observe a significant association between race and treatment with MRA which persisted after adjustment. With respect to target dosing, after adjustment for demographic characteristics, race did not independently predict BB target dosing; however, the association between race and target treatment with ACEi/ARB/ARNi and MRA persisted. The reasons for these differences are not immediately clear. It is possible that our results may reflect provider characteristics or trends in clinical decision‐making processes which are not fully captured in our model, and which may contribute to the higher rates and target treatment for these classes in Black patients. Prior studies have referenced the ideas of clinical inertia, financial toxicity, lack of standardization of healthcare, and structural racism as contributing to impaired GDMT uptake in racially diverse populations [6]. Further investigation is necessary to fully understand and address the underlying reasons for the increased MRA utilization, and higher ACEi/ARB/ARNi and MRA target dosing among Black patients, ensuring equitable and optimized treatment across all GDMT classes.

Black patients have been reported as less likely than non‐Black patients to receive guideline‐directed cardiovascular diagnostic and therapeutic interventions as well as being less likely to be admitted to cardiology specialty services, receive cardiology specialty care, and be referred for cardiac transplant [29, 30, 31, 32, 33, 34, 35]. Our results revealed similarly decreased healthcare utilization, including fewer cardiology visits and number of hospitalizations among Black patients. Black patients have been underrepresented in pivotal HF clinical trials [36], so our finding that Black patients are more likely to receive certain classes of GDMT medications is interesting and points to the importance of documenting GDMT treatment patterns by race in real‐world data. It is all the more noteworthy that, despite these other reported racial gaps in cardiac care for Black patients including higher risks of HF, HF hospitalization, and HF‐related death [6, 7, 8], GDMT use and target dosing across certain classes appear to be more likely among Black patients than among White counterparts in this particular health system population. The reasons behind the higher treatment and target treatment rates among Black patients—and, notably, the contrast to other care and utilization disparities seen in this minority group—warrant further study.

Several limitations of our analysis warrant mention. First, our study was observational, and thus causal relationships could not be established. Second, because we used fill data instead of order data to assess use and doses of GDMT medications, our data may not capture actual provider intent nor true patient medication adherence. Actual medication prescribing may be higher than reflected in our data of medication use. Third, patients with missing covariate data were excluded from the analysis, and this complete case analysis may have introduced bias into our results by potentially omitting systematic differences between included and excluded patients. Fourth, because our population was a predominantly insured population within a single US healthcare system, the generalizability of our findings to broader US or global populations is limited.

Despite these limitations, our study had notable strengths including the comprehensive EHR data, which provided not only standard demographic and claims data but also detailed pharmacy data, clinical measures, laboratory, and healthcare utilization data. Additionally, our diverse sample of majority Black patients allowed us to provide real‐world data for this typically underrepresented population.

## 5 Conclusion

In our study of patients with HFrEF, several racial differences in recommended GDMT treatment were identified. We found that Black patients were more likely to be treated with MRAs than White patients. Among those treated with any GDMT, Black patients were more likely to be treated to target with ACEi/ARB/ARNis and MRAs compared with White patients. This analysis adds to the existing literature comparing GDMT rates and doses in Black versus White patients in a large, diverse, real‐world HFrEF patient population with robust representation from Black patients. Ultimately, while these proposed insights into the observed differences in racial GDMT treatment are plausible, the complexity of treatment inequities in HF extends far beyond the scope of this study. Future research should explore in greater detail the causes of racial differences in GDMT and determine whether they result in meaningful clinical differences in health outcomes for Black patients with HFrEF.

## Author Contributions

**Natalia C. Berry:** conceptualization, data curation, formal analysis, investigation, methodology, writing – original draft. **Yi‐Shin Sheu:** data curation, formal analysis, investigation, methodology, writing – review & editing. **Karen Chesbrough:** investigation, project administration, writing – review & editing. **R. Clayton Bishop:** visualization, writing – original draft. **Suma Vupputuri:** conceptualization, formal analysis, investigation, methodology, supervision, writing – original draft. All authors read and approved the manuscript.

## Funding

The authors have nothing to report.

## Ethics Statement

This study was approved by the Kaiser Permanente Mid‐Atlantic States Institutional Review Board with a waiver of informed consent.

## Conflicts of Interest

The authors declare no conflicts of interest.

## Supporting information


Supporting File

## Data Availability

The data that support the findings of this study are available on request from the corresponding author. The data are not publicly available due to privacy or ethical restrictions.
