# Supplementary material for: Comparison of Guideline‐Directed Medical Therapy in Black versus White Patients With Heart Failure With Reduced Ejection Fraction in a Large, Integrated Health Care System
Source: Clin Cardiol. 2026 Aug 30;49(9):e70452. doi: 10.1002/clc.70452 (PMC13527225; doi:10.1002/clc.70452)
Supplement: Supplementary file 1 [file CLC-49-e70452-s001.docx]

**Supplementary Information**

**Comparison of Guideline-Directed Medical Therapy in Black Versus White Patients with Heart Failure with Reduced Ejection Fraction in a Large, Integrated Health Care System**

**Supplemental Methods**

**CHF Diagnosis Definition:**

CHF was established by examining inpatient and outpatient encounters, as well as ICD-9/10 codes where appropriate:

A diagnosis of HF is based on either having been previously hospitalized with a principal discharge diagnosis of HF and/or having ≥3 ambulatory visits coded for HF based on International Classification of Diseases-9th Edition (398.91, 402.x1, 404.01, 404.03, 404.11, 404.13, 404.91, 404.93, and 428.x) and -10th Edition (I09.81, I11.0, I11.9, I13.0, I13.1, I13.10, I13.11, I13.2, I50, I50.1, I50.2, I50.20, I50.21, I50.22, I50.23, I50.3, I50.30, I50.31, I50.32, I50.33, I50.4, I50.40, I50.41, I50.42, I50.43, I50.9, and I97.13) codes. These codes have been validated in multiple health care delivery systems and have a positive predictive value ≥95%.

**Supplementary Table 1. Patient data**

| **Demographics** | **Disease history** | **Cardiology-related encounters or procedures** | **Vital signs** | **Labs** |
| --- | --- | --- | --- | --- |
| Age at index date | Atrial fibrillation or flutter | Cardiology encounter | Body mass index | HDL |
| Sex | Ischemic stroke or TIA | Primary care physician encounter | Systolic blood pressure | LDL |
| Race | Coronary artery disease | Echocardiogram | Heart rate (bpm) | Hemoglobin |
| Ethnicity | Hypertension | Heart failure hospitalization within 12 months |  | Hemoglobin A1C |
| Neighborhood deprivation index | Dyslipidemia/hyperlipidemia | Cardiac catheterization |  | Albumin |
| Spoken language | Hyperthyroidism | cardiac resynchronization therapy |  | BNP |
|  | Hypothyroidism | Automated implantable defibrillator |  |  |
|  | Chronic liver disease/cirrhosis |  |  |  |
|  | Asthma |  |  |  |
|  | COPD |  |  |  |
|  | Home Oxygen therapy |  |  |  |
|  | Depression |  |  |  |
|  | Dementia/Cognitive conditions |  |  |  |
|  | left ventricular ejection fraction |  |  |  |
|  | Current or prior smoker |  |  |  |
|  | Chronic Renal Failure |  |  |  |

**Supplementary Table 2: GDMT Target dose table** (Ref: Heidenreich, P.A., et al., *2022 AHA/ACC/HFSA Guideline for the Management of Heart Failure: A Report of the American College of Cardiology/American Heart Association Joint Committee on Clinical Practice Guidelines.* Circulation, 2022. **145**(18): p. e895-e1032.)

**Supplementary Table 3: Contraindications to GDMT**

| **Medication** | **Contraindication** | **Time Period** |
| --- | --- | --- |
| ACEi/ARB/ARNi | CKD stage V N18.5 (ICD10), 585.5, (ICD9) | Within 12 months prior to index |
| Beta Blockers | None | N/A |
|  |  |  |
| Mineralocorticoid Receptor Antagonists | Hyperkalemia E87.5 (ICD 10), 267.7 (ICD 9)  CKD stage V N18.5 (ICD10), 585.5 (ICD9) | Within 12 months prior to index  Within 12 months prior to index |
| SGLT2i | CKD stage IV or V, N18.5, N18.4 (ICD10), 585.5, 585.4 (ICD9) | Within 12 months prior to index |

**Supplementary Table 4. Odds ratios for covariates in model of association of race with GDMT treatment**

|  | **GDMT Class** | | |
| --- | --- | --- | --- |
|  | **ACEi/ARB/ARNi** | **BB** | **MRA** |
|  | **OR (95% CI)** | **OR (95% CI)** | **OR (95% CI)** |
| Age | 0.99 **(0.98, 1.00)** | 0.96 **(0.95, 0.97)** | 0.98 **(0.97, 0.99)** |
| Sex | 0.91 (0.73, 1.15) | 1.05 (0.84, 1.30) | 0.82 **(0.67, 1.00)** |
| Race | 1.14 (0.88, 1.47) | 1.20 (0.94, 1.53) | 1.39 **(1.10, 1.75)** |
| Neighborhood Deprivation Index |  |  |  |
| 2^nd^ Quartile | 1.15 (0.84, 1.58) | 0.94 (0.71, 1.25) | 1.11 (0.83, 1.47) |
| 3^rd^ Quartile | 0.90 (0.66, 1.23) | 1.12 (0.83, 1.52) | 1.10 (0.83, 1.48) |
| 4^th^ Quartile | 0.94 (0.68, 1.30) | 1.22 (0.89, 1.68) | 1.40 (1.05, 1.87) |
|  |  |  |  |
| Left ventricular ejection fraction | 0.98 **(0.97, 1.00)** | 0.97 **(0.96, 0.98)** | 0.98 **(0.97, 0.99)** |
| History of atrial fibrillation or flutter | 0.71 **(0.56, 0.89)** | 0.66 **(0.54, 0.82)** | 0.80 **(0.65, 1.00)** |
| History of ischemic stroke or transient ischemic attack | 0.91 (0.72, 1.14) | 0.83 (0.67, 1.03) | 0.74 **(0.60, 0.90)** |
| History of coronary artery disease | 0.90 (0.71, 1.16) | 0.95 (0.75, 1.20) | 1.06 (0.86, 1.32) |
| Hypertension | 1.12 (0.81, 1.55) | 1.20 (0.88, 1.63) | 1.31 **(1.00, 1.72)** |
| Dyslipidemia/hyperlipidemia | 1.35 **(1.05, 1.75)** | 1.02 (0.79, 1.31) | 1.14 (0.92, 1.43) |
| Chronic liver disease/cirrhosis | 0.88 (0.64, 1.19) | 0.91 (0.67, 1.23) | 1.10 (0.83, 1.46) |
| Asthma | 0.85 (0.63, 1.15) | 1.09 (0.81, 1.47) | 0.89 (0.68, 1.16) |
| Chronic obstructive pulmonary disease | 0.72 **(0.57, 0.92)** | 0.84 (0.67, 1.06) | 0.98 (0.78, 1.23) |
| Home oxygen therapy | 0.44 **(0.28, 0.71)** | 0.63 (0.39, 1.02) | 1.15 (0.67, 1.98) |
| Depression | 0.82 (0.63, 1.07) | 0.79 (0.61, 1.01) | 0.94 (0.73, 1.21) |
| Dementia/cognitive conditions | 0.76 (0.56, 1.01) | 0.73 **(0.55, 0.96)** | 0.92 (0.68, 1.25) |
| Smoker | 0.87 (0.65, 1.15) | 0.96 (0.72, 1.27) | 0.89 (0.69, 1.13) |
|  |  |  |  |
| Systolic blood pressure | 1.02 **(1.01, 1.02)** | 1.00 (0.99, 1.01) | 1.00 (0.99, 1.00) |
| Body mass index | 1.02 (1.00, 1.03) | 0.99 (0.98, 1.01) | 1.02 **(1.01, 1.04)** |
| Hemoglobin A1C |  |  |  |
| High | 1.23 (0.87, 1.73) | 0.96 (0.70, 1.34) | 1.07 (0.79, 1.45) |
| Elevated | 0.93 (0.66, 1.31) | 0.89 (0.64, 1.24) | 1.07 (0.78, 1.46) |
| Unknown | 1.05 (0.74, 1.48) | 0.98 (0.71, 1.37) | 0.96 (0.70, 1.30) |
| Estimated glomerular filtration rate (eGFR) |  |  |  |
| Severe | 0.16 **(0.10, 0.24)** | 0.87 (0.54, 1.38) | 0.21 **(0.10, 0.44)** |
| Moderate | 0.58 **(0.46, 0.73)** | 0.98 (0.78, 1.23) | 0.72 **(0.58, 0.90)** |
| Unknown | 1.21 (0.40, 3.64) | 1.13 (0.38, 3.41) | 1.21 (0.51, 2.83) |
|  |  |  |  |
| Cardiology visits, 12-month period | 1.00 (0.99, 1.01) | 1.00 (0.99, 1.00) | 1.01 **(1.00, 1.02)** |
| Primary care visits, 12-month period | 1.00 (0.99, 1.00) | 1.00 (1.00, 1.01) | 1.00 (1.00, 1.01) |
| Inpatient hospitalizations, 12-month period | 0.92 (0.80, 1.05) | 1.06 (0.91, 1.23) | 1.01 (0.87, 1.17) |
| Automated implantable defibrillator | 1.24 (0.85, 1.81) | 2.05 **(1.37, 3.09)** | 1.51 **(1.10, 2.08)** |

**Supplementary Table 5. Odds ratios for covariates in model of association of race with GDMT target dosing**

|  | **GDMT Class** | | |
| --- | --- | --- | --- |
|  | **ACEi/ARB/ARNi** | **BB** | **MRA** |
|  | **OR (95% CI)** | **OR (95% CI)** | **OR (95% CI)** |
| Age | 1.01 (1.00, 1.02) | 0.98 **(0.97, 0.99)** | 1.01 (0.99, 1.03) |
| Sex | 1.17 (0.96, 1.42) | 1.17 (0.93, 1.47) | 1.09 (0.72, 1.64) |
| Race | 2.01 **(1.60, 2.53)** | 1.23 (0.94, 1.62) | 1.61 **(1.01, 2.57)** |
| Neighborhood Deprivation Index | 1.19 (0.91, 1.57) | 1.19 (0.86, 1.65) | 1.13 (0.62, 2.06) |
| 2^nd^ Quartile |  |  |  |
| 3^rd^ Quartile | 1.01 (0.76, 1.33) | 1.05 (0.75, 1.46) | 0.95 (0.52, 1.74) |
| 4^th^ Quartile | 1.15 (0.86, 1.53) | 1.34 (0.96, 1.87) | 0.83 (0.46, 1.48) |
|  |  |  |  |
| Left ventricular ejection fraction | 1.02 **(1.01, 1.03)** | 1.01 **(1.00, 1.03)** | 1.01 (0.98, 1.03) |
| History of atrial fibrillation or flutter | 0.81 **(0.65, 0.99)** | 1.40 **(1.10, 1.78)** | 0.92 (0.59, 1.46) |
| History of ischemic stroke or transient ischemic attack | 0.76 **(0.62, 0.93)** | 1.07 (0.85, 1.35) | 1.25 (0.82, 1.92) |
| History of coronary artery disease | 0.77 **(0.63, 0.96)** | 0.86 (0.67, 1.10) | 0.92 (0.58, 1.46) |
| Hypertension | 1.23 (0.94, 1.61) | 1.03 (0.75, 1.40) | 1.03 (0.58, 1.82) |
| Dyslipidemia/hyperlipidemia | 1.32 **(1.06, 1.65)** | 1.20 (0.93, 1.54) | 0.64 (0.39, 1.04) |
| Chronic liver disease/cirrhosis | 0.99 (0.75, 1.32) | 0.63 **(0.44, 0.89)** | 1.54 (0.83, 2.85) |
| Asthma | 1.15 (0.88, 1.52) | 0.61 **(0.44, 0.84)** | 1.02 (0.56, 1.84) |
| Chronic obstructive pulmonary disease | 1.01 (0.81, 1.26) | 1.10 (0.85, 1.41) | 1.02 (0.63, 1.65) |
| Home oxygen therapy | 0.72 (0.38, 1.37) | 1.49 (0.80, 2.78) | 1.34 (0.40, 4.46) |
| Depression | 1.17 (0.92, 1.50) | 1.10 (0.83, 1.47) | 1.10 (0.66, 1.85) |
| Dementia/cognitive conditions | 0.79 (0.59, 1.06) | 0.75 (0.53, 1.07) | 1.00 (0.52, 1.92) |
| Smoker | 0.98 (0.77, 1.26) | 0.80 (0.60, 1.06) | 1.08 (0.65, 1.80) |
|  |  |  |  |
| Systolic blood pressure | 1.05 **(1.05, 1.06)** | 1.03 **(1.02, 1.03)** | 1.00 (0.99, 1.02) |
| Body mass index | 1.02 **(1.01, 1.04)** | 1.02 **(1.01, 1.04)** | 1.02 (0.99, 1.05) |
| Hemoglobin A1C |  |  |  |
| High (≥6.5%) | 1.17 (0.87, 1.57) | 1.63 **(1.14, 2.33)** | 1.21 (0.63, 2.33) |
| Elevated (5.7-6.4%) | 0.90 (0.66, 1.23) | 1.37 (0.94, 2.00) | 0.84 (0.43, 1.62) |
| Unknown | 0.72 **(0.53, 0.98)** | 1.34 (0.92, 1.94) | 0.78 (0.41, 1.50) |
| Estimated glomerular filtration rate (eGFR) |  |  |  |
| Severe (<30 mL/min/1.73m^2^) | 0.96 (0.53, 1.75) | 1.57 (0.96, 2.57) | 0.53 (0.11, 2.55) |
| Moderate (30-60 mL/min/1.73m^2^) | 1.07 (0.86, 1.31) | 1.11 (0.88, 1.41) | 0.77 (0.49, 1.20) |
| Unknown | 0.68 (0.26, 1.74) | 0.75 (0.26, 2.14) | 0.77 (0.14, 4.26) |
|  |  |  |  |
| Cardiology visits, 12-month period | 1.01 (1.00, 1.01) | 1.01 (1.00, 1.01) | 1.00 (0.98, 1.01) |
| Primary care visits, 12-month period | 1.00 (1.00, 1.01) | 1.00 (0.99, 1.00) | 0.99 (0.98, 1.00) |
| Inpatient hospitalizations, 12-month period | 1.02 (0.88, 1.18) | 0.97 (0.83, 1.14) | 1.01 (0.73, 1.38) |
| Automated implantable defibrillator | 2.03 **(1.45, 2.85)** | 2.84 **(2.01, 4.02)** | 0.82 (0.45, 1.50) |
